# Assessment of the Role of Artificial Intelligence in the Association Between Time of Day and Colonoscopy Quality

**DOI:** 10.1001/jamanetworkopen.2022.53840

**Published:** 2023-01-31

**Authors:** Zihua Lu, Lihui Zhang, Liwen Yao, Dexin Gong, Lianlian Wu, Meiqing Xia, Jun Zhang, Wei Zhou, Xu Huang, Chunping He, Huiling Wu, Chenxia Zhang, Xun Li, Honggang Yu

**Affiliations:** 1 Department of Gastroenterology, Renmin Hospital of Wuhan University, Wuhan, China; 2 Hubei Provincial Clinical Research Center for Digestive Disease Minimally Invasive Incision, Renmin Hospital of Wuhan University, Wuhan, China; 3 Key Laboratory of Hubei Province for Digestive System Disease, Renmin Hospital of Wuhan University, Wuhan, China; 4 Department of Gastroenterology, Wuhan Jiangxia District Hospital of Traditional Chinese Medicine, Wuhan, China

## Abstract

**Importance:**

Time of day was associated with a decline in adenoma detection during colonoscopy. Artificial intelligence (AI) systems are effective in improving the adenoma detection rate (ADR), but the performance of AI during different times of the day remains unknown.

**Objective:**

To validate whether the assistance of an AI system could overcome the time-related decline in ADR during colonoscopy.

**Design, Setting, and Participants:**

This cohort study is a secondary analysis of 2 prospective randomized controlled trials (RCT) from Renmin Hospital of Wuhan University. Consecutive patients undergoing colonoscopy were randomly assigned to either the AI-assisted group or unassisted group from June 18, 2019, to September 6, 2019, and July 1, 2020, to October 15, 2020. The ADR of early and late colonoscopy sessions per half day were compared before and after the intervention of the AI system. Data were analyzed from March to June 2022.

**Exposure:**

Conventional colonoscopy or AI-assisted colonoscopy.

**Main Outcomes and Measures:**

Adenoma detection rate.

**Results:**

A total of 1780 patients (mean [SD] age, 48.61 [13.35] years, 837 [47.02%] women) were enrolled. A total of 1041 procedures (58.48%) were performed in early sessions, with 357 randomized into the unassisted group (34.29%) and 684 into the AI group (65.71%). A total of 739 procedures (41.52%) were performed in late sessions, with 263 randomized into the unassisted group (35.59%) and 476 into the AI group (64.41%). In the unassisted group, the ADR in early sessions was significantly higher compared with that of late sessions (13.73% vs 5.70%; *P* = .005; OR, 2.42; 95% CI, 1.31-4.47). After the intervention of the AI system, as expected, no statistically significant difference was found (22.95% vs 22.06%, *P* = .78; OR, 0.96; 95% CI; 0.71-1.29). Furthermore, the AI systems showed better assistance ability on ADR in late sessions compared with early sessions (odds ratio, 3.81; 95% CI, 2.10-6.91 vs 1.60; 95% CI, 1.10-2.34).

**Conclusions and Relevance:**

In this cohort study, AI systems showed higher assistance ability in late sessions per half day, which suggests the potential to maintain high quality and homogeneity of colonoscopies and further improve endoscopist performance in large screening programs and centers with high workloads.

## Key Points

**Question:**

Can artificial intelligence (AI) systems eliminate time-related declines in adenoma detection during colonoscopy?

**Findings:**

In this cohort study including 1780 colonoscopy procedures, in the unassisted group, the adenoma detection rate (ADR) at late sessions was significantly higher compared with that of early sessions, while with the assistance of AI systems, no such statistically significant difference was found.

**Meaning:**

These findings suggest AI systems may be a potential tool for minimizing time-related degradation of colonoscopy quality.

## Teaser

This cohort study evaluates the role of artificial intelligence in the association between time of day and colonoscopy quality.

## Introduction

Colorectal cancer (CRC) is the third most common cancer worldwide.^1^ Prevention strategies—especially screening colonoscopy—can substantially reduce CRC incidence and mortality via detection and removal of precancerous polyps.^2,3^ Adenoma detection rate (ADR) is a critical quality measure of screening colonoscopy and each 1.0% increase in ADR is associated with a 3.0% decrease in the risk of interval CRC (hazard ratio, 0.97; 95% CI, 0.96-0.98).^4^ Ensuring high ADR is hence paramount in the prevention of CRC.

Time of day has been identified as an indispensable factor related to suboptimal ADR. Sanaka et al^5^ first reported performance time of the day as an independent risk factor of colonoscopy quality. Subsequently, substantial evidence has shown that morning colonoscopies are associated with improved ADR.^6,7,8,9^ According to a previous study,^7^ the afternoon ADR was significantly lower than in the morning (21.0% vs 26.1%; *P* = .02) for endoscopists working the full day. In addition, Lee et al^10^ reported that each elapsed hour in the day was associated with a 4.6% reduction in polyp detection (*P* = .005). Continuous and repetitive visual stimuli may lead to weaker reliable response and poorer judgment, which jeopardize diagnostic abilities and efficiency. Unfortunately, an objective approach to minimize time-related degradation of colonoscopy quality is still lacking.

According to available evidence, the incorporation of artificial intelligence (AI) as an aid for colonoscopy results in a significant increase in ADR.^11^ There are 2 main reasons for missed polyps: polyps that are unrecognized inside the visual field due to repetitive movement and monotonous vision and missed polyps outside the visual field because endoscopists are more rushed through the course of the day, so blind spots increase during the withdrawal phase.^12,13^ In our previous studies, we have developed a deep-learning based computer-aided detection (CADe) system to augment detection of polyps inside the visual field and a computer-aided quality improvement (CAQ) system to improve withdrawal visualization quality.^14,15^ Through randomized clinical trials (RCT), we have demonstrated its positive association with increasing ADR.^14,15^ However, the extra benefit of AI systems in eliminating the time-related decline of ADR remains unknown.

Therefore, we hypothesized that AI systems may be an effective tool to address the previously stated problem. The aim of this study was to validate whether the assistance of AI systems could overcome the time-related degradation of colonoscopy quality according to the data from our previous randomized clinical trials.

## Method

### Study Cohort

This study is a secondary analysis of 2 RCTs. Patients undergoing screening, diagnosis, and surveillance colonoscopy were included in this study, but not those undergoing therapeutic colonoscopy. Details of the interventions are provided in the eMethods in Supplement 1. RCT 1 was a prospective, randomized parallel group study to evaluate whether the CAQ system could improve ADR during colonoscopy. Consecutive patients who were able to give informed consent were randomly assigned (1:1) to either the CAQ colonoscopy group (355 patients) or the unassisted (control) colonoscopy group (349 patients) between June 18, 2019, and September 6, 2019. The random allocation sequence was a computer-generated random number series, and randomization was done in blocks of 4. All the colonoscopies were performed by 6 endoscopists (including J.Z. and X.H.) with endoscopy experience of 1 to 3 years and a total colonoscopy volume of 1500 to 4000.^14^ RCT 2 was a prospective, single-center, 4-group, randomized parallel study to evaluate the interaction effect of improving the ADR between a CADe system for colorectal polyp detection and a CAQ system for real-time withdrawal speed monitoring. Between July 1, 2020, and October 15, 2020, 1076 participants were randomly allocated into 4 treatment groups (control: 271; CADe: 268; CAQ: 269; and CADe plus CAQ [combination]: 268). An electronic digital capture system was used to generate a random number of each patient before the procedure, and randomization was done in blocks of 16. All the colonoscopies were performed by 4 endoscopists (L.W., J.Z., W.Z., and C.H.) with more than 2000 screening colonoscopies in experience.^15^ The ethics committees of Renmin Hospital of Wuhan University approved both protocols, and all participants provided written informed consent. We followed the Strengthening the Reporting of Observational Studies in Epidemiology (STROBE) reporting guideline.

### Definition

In this study, the AI group was defined as the combination of all intervention groups, including the CAQ group in RCT 1 and the CAQ group, CADe group, and combination group in RCT 2. The combined control (control-c) group was defined as the combination of control groups in RCT 1 and RCT 2. For single computer-aided functions, the combined CAQ (CAQ-c) group was defined as the combination of the CAQ group in RCT 1 and the CAQ group in RCT 2.

Full-day colonoscopy procedures were included in this study. A half day was defined as morning (earlier than 1 pm) and afternoon (1 pm or later) according to previous studies.^16^ Since this study was focused on the process of withdrawal, to avoid the influence of long-time insertion on the research group, we used the end time of the procedure to define early and late sessions, which is closer to the process of examination. All colonoscopy procedures were divided into 2 groups according to the end time of procedure. The first group consisted of procedures commenced in the early session per half day (8:00 am to 10:59 am or 1:00 pm to 2:59 pm), which was defined as the early group, and the second group consisted of procedures commenced in the later session per half day (11:00 am to 12:59 pm or 3:00 pm to 4:59 pm), which was defined as the late group. We divided endoscopists into 3 groups according to their experience, high experience (≥3000 colonoscopies), middle experience (2000-3000 colonoscopies), and low experience (<2000 colonoscopies). Detailed definitions of ADR, polyp detection rate (PDR), proximal colon, distal colon and advanced adenoma were provided in the eMethods in Supplement 1.

### Statistical Analysis

Baseline characteristics are reported with early sessions and late sessions. Continuous variables were reported as mean with SD, while categorical variables were reported as frequencies and percentages. Comparison of baseline clinical and demographic characteristics between the early and the late session was performed using a χ^2^ test for categorical variables and using a 2-sample *t* test for continuous variables. Logistic regression was applied to assess the association between the intervention and the ADR/PDR. A covariate-adjusted logistic regression model was built by adding group differences into the models as covariates to address possible confounding factors, including withdrawal time, endoscopist experience, bowel preparation, age, sex, body mass index (BMI; calculated as weight in kilograms divided by height in meters squared), indication for colonoscopy, inpatient or outpatient status, and endoscope vendors. A Poisson regression was performed to assess the association between the intervention and the mean number of adenomas and polyps per colonoscopy. Odds ratios (ORs) with 95% CIs and corresponding *P* values were calculated. All reported *P* values are 2-sided, and *P* values of less than .05 were considered statistically significant. All calculations were performed using SPSS version 26 (IBM). Data were analyzed from March to June 2022.

## Results

### Characteristics of the Study Cohort

A total of 1780 patients (mean [SD] age, 48.61 [13.35] years, 837 [47.02%] women) were enrolled. Overall, 58.48% of procedures (1041 of 1780) were performed in early sessions, with 34.29% (357 of 1041) randomized into the control-c group and 65.71% (684 of 1041) into the AI group. A total of 41.52% of procedures (739 of 1780) were performed in late sessions, with 35.59% (263 of 739) randomized into the control-c group and 64.41% (476 of 739) into the AI group. There were 863 polyps and 326 adenomas detected in the study cohort. Baseline characteristics are presented in Table 1.

**Table 1.** Baseline Characteristics

Characteristic	Patients, No. (%)		*P* value
	Early session (n = 1041)	Late session (n = 739)	
Age, mean (SD), y	50.14 (13.21)	48.53 (13.59)	.01
Sex			
Male	497 (47.74)	340 (46.01)	.47
Female	544 (52.26)	399 (53.99)	
Body mass index^a^			
<25	791 (75.98)	594 (80.38)	.05
25 to <30	223 (21.42)	124 (16.78)	
≥30	27 (2.59)	21 (2.84)	
Indication of colonoscopy			
Screening	670 (64.36)	414 (56.02)	<.001
Diagnostic	283 (27.19)	276 (37.35)	
Surveillance	88 (8.45)	49 (6.63)	
Inpatient	190 (18.25)	158 (21.38)	.10
Outpatient	851 (81.75)	581 (78.62)	
Endoscope vendor			
Olympus	686 (65.90)	482 (65.22)	.09
Fujifilm	304 (29.20)	203 (27.47)	
Sonoscape	51 (4.90)	54 (7.31)	
Withdrawal time, mean (SD), min	8.21 (4.33)	7.81 (4.38)	.07
Bowel preparation			
Boston bowel preparation scale			
<2 In any segment	125 (12.01)	80 (10.83)	.44
≥2 In all segments	916 (87.99)	659 (89.17)	
Endoscopist experience			
High experience	465 (44.67)	347 (46.96)	.42
Mid experience	472 (45.34)	312 (42.22)	
Low experience	104 (9.99)	80 (10.83)	

^a^ Body mass index is calculated as weight in kilograms divided by height in meters squared.

### Association of Time of Day and ADR/PDR

In the control-c group, the ADR at early sessions was significantly higher compared with that of late sessions (early vs late, 13.73% vs 5.70%; *P* = .005; OR, 2.42; 95% CI, 1.31-4.47). After the intervention of the AI system, as expected, no statistically significant difference was found in ADR between early and late sessions (early vs late, 22.95% vs 22.06%; *P* = .78; OR, 0.96; 95% CI; 0.71-1.29) or PDR (early vs late, 55.99% vs 52.31%; *P* = .57; OR, 1.08; 95% CI, 0.83-1.40). Also, the CADe group (early vs late, 25.30% vs 14.71%; *P* = .08; OR, 1.97; 95% CI, 0.93-4.17), CAQ-c group (early vs late, 19.48% vs 20.00%; *P* = .69; OR, 0.92; 95% CI, 0.60-1.39), and combination group (early vs late, 27.81% vs 35.35%; *P* = .13; OR, 0.63; 95% CI, 0.34-1.15) showed no statistically significant difference in adenoma detection between early and late sessions. Subgroup analysis of different bowel preparation, withdrawal time, and endoscopist experience showed the same association (Table 2). Other detection rates, such as advanced adenoma and analysis in different RCTs, are shown in eTable 1 and eTable 2 in Supplement 1. Detailed analysis among individual endoscopists who performed more than 150 colonoscopies in this study is shown in eTable 3 in Supplement 1.

**Table 2.** Comparison of Detection Rates Between Early and Late Session

Characteristic	ADR, No. (%)		*P* value	OR (95% CI)	PDR, No. (%)		*P* value	OR (95% CI)
	Early	Late			Early	Late		
Unassisted group	49 (13.73)	15 (5.70)	.005	2.42 (1.31-4.47)	148 (41.46)	83 (31.56)	.12	1.34 (0.93-1.92)
BBPS								
≥2 In all segments	45 (14.24)	14 (5.81)	.006	2.45 (1.29-4.66)	128 (40.51)	75 (31.12)	.14	1.34 (0.91-1.95)
<2 In any segment	4 (9.76)	1 (4.55)	.35	4.71 (0.18-124.63)	20 (48.78)	8 (36.36)	.96	0.97 (0.25-3.83)
Withdrawal time, min								
≥6	33 (17.46)	8 (7.08)	.02	2.68 (1.17-6.14)	92 (48.68)	45 (39.82)	.32	1.29 (0.78-2.15)
<6	16 (9.52)	7 (4.67)	.14	2.03 (0.79-5.25)	56 (33.33)	38 (25.33)	.14	1.51 (0.88-2.59)
Endoscopist experience								
High experience	9 (16.07)	5 (12.20)	.52	1.51 (0.43-5.25)	29 (51.79)	14 (34.15)	.22	1.85 (0.70-4.91)
Mid experience	19 (15.08)	3 (3.06)	.006	6.42 (1.71-24.10)	47 (37.30)	27 (27.55)	.41	1.30 (0.69-2.45)
Low experience	21 (12.00)	7 (5.65)	.18	1.88 (0.75-4.72)	72 (41.14)	42 (33.87)	.49	1.20 (0.71-2.01)
AI group	157 (22.95)	105 (22.06)	.78	0.96 (0.71-1.29)	383 (55.99)	249 (52.31)	.57	1.08 (0.83-1.40)
CADe	42 (25.30)	15 (14.71)	.08	1.97 (0.93-4.17)	102 (61.45)	47 (46.08)	.12	1.58 (0.89-2.81)
Combined CAQ group	68 (19.48)	55 (20.00)	.69	0.92 (0.60-1.39)	171 (49.00)	140 (50.91)	.52	0.89 (0.63-1.26)
Combined CADe and CAQ group	47 (27.81)	35 (35.35)	.13	0.63 (0.34-1.15)	110 (65.09)	62 (62.63)	.65	1.15 (0.63-2.08)
BBPS								
≥2 In all segments	135 (22.50)	91 (21.77)	.63	0.93 (0.67-1.27)	345 (57.50)	219 (52.39)	.41	1.12 (0.85-1.48)
<2 In any segment	22 (26.19)	14 (24.14)	.50	1.35 (0.56-3.26)	38 (45.24)	30 (51.72)	.48	0.76 (0.36-31.63)
Withdrawal time, min								
≥6	144 (25.90)	91 (23.51)	.88	1.02 (0.74-1.41)	336 (60.43)	215 (55.56)	.51	1.10 (0.83-1.47)
<6	13 (10.16)	14 (15.73)	.28	0.62 (0.26-1.48)	47 (36.72)	34 (38.20)	.99	1.00 (0.54-1.85)
Endoscopist experience								
High experience	6 (12.50)	12 (30.77)	.06	0.34 (0.11-1.06)	19 (39.58)	22 (56.41)	.21	0.55 (0.22-1.39)
Mid experience	91 (26.30)	47 (21.96)	.43	1.20 (0.77-1.86)	200 (57.80)	109 (50.93)	.29	1.23 (0.84-1.81)
Low experience	60 (20.69)	46 (20.63)	.74	0.93 (0.58-1.47)	164 (56.55)	118 (52.91)	.80	1.05 (0.71-1.56)

Abbreviations: ADR, adenoma detection rate; BBPS, Boston bowel preparation scale; CADe, computer-aided detection; CAQ, computer-aided quality improvement; OR, odds ratio; PDR, polyp detection rate.

### Association of Time of Day and AI Assistance Capability

The assistance capability of AI systems was compared through OR values.^17^ For adenoma detection, the ADR was significantly improved after the assistance of AI systems in both early sessions (22.95% vs 13.73%; *P* = .01; OR, 1.60; 95% CI, 1.10-2.34) and late sessions (22.06% vs 5.70%; *P* < .001; OR, 3.81; 95% CI, 2.10-6.91). Furthermore, the AI systems had higher assistance capability in late sessions compared with early sessions (OR, late vs early, 3.81; 95% CI, 2.10-6.91 vs 1.60; 95% CI, 1.10-2.34). Subgroup analysis of different bowel preparation, withdrawal time, and endoscopist experience showed the same trend (Table 3).

**Table 3.** Comparison of AI Assistance Capabilities Between Early and Late Session

Outcome	No.	Early session			Late session		
		Detection rate, AI vs unassisted, %	*P* value	OR (95% CI)	Detection rate, AI vs unassisted, %	*P* value	OR (95% CI)
**Adenoma detection rate**							
All procedures	1780	22.95 vs 13.73	.01	1.60 (1.10-2.34)	22.06 vs 5.70	<.001	3.81 (2.10-6.91)
BBPS							
≥2 In all segments	1575	22.50 vs 14.24	.07	1.46 (0.98-2.17)	21.77 vs 5.81	<.001	3.63 (1.94-6.76)
<2 In any segment	205	26.19 vs 9.76	.08	3.39 (0.89-12.98)	24.14 vs 4.55	.10	9.06 (0.66-123.65)
Withdrawal time, min							
≥6	1245	25.90 vs 17.46	.006	1.85 (1.19-2.87)	23.51 vs 7.08	<.001	4.24 (1.95-9.19)
<6	535	10.16 vs 9.52	.79	1.12 (0.48-2.61)	15.73 vs 4.67	.02	3.41 (1.26-9.23)
Endoscopist experience							
High experience	184	12.50 vs 16.07	.80	0.86 (0.26-2.85)	30.77 vs 12.20	.12	2.65 (0.78-8.97)
Mid experience	784	26.30 vs 15.08	.03	1.91 (1.07-3.42)	21.96 vs 3.06	.003	6.32 (1.85-21.58)
Low experience	812	20.69 vs 12.00	.25	1.41 (0.79-2.54)	20.63 vs 5.65	.001	4.03 (1.62-10.02)
**Polyp detection rate**							
All procedures	1780	55.99 vs 41.46	.005	1.53 (1.14-2.06)	52.31 vs 31.56	.001	1.90 (1.32-2.73)
BBPS							
≥2 In all segments	1575	57.50 vs 40.51	.001	1.67 (1.22-2.28)	52.39 vs 31.12	<.001	1.99 (1.36-2.93)
<2 In any segment	205	45.24 vs 48.78	.54	0.73 (0.27-1.98)	51.72 vs 36.36	.99	1.01 (0.28-3.61)
Withdrawal time							
≥6	1245	60.43 vs 48.68	.001	1.87 (1.30-2.69)	55.56 vs 39.82	.004	1.97 (1.24-3.10)
<6	535	36.72 vs 33.33	.75	1.09 (0.64-1.88)	38.20 vs 25.33	.04	2.02 (1.04-3.91)
Endoscopist experience							
High experience	184	39.58 vs 51.79	.38	0.68 (0.29-1.62)	56.41 vs 34.15	.10	2.59 (0.84-7.95)
Mid experience	784	57.80 vs 37.30	.002	2.10 (1.31-3.37)	50.93 vs 27.55	.04	1.91 (1.04-3.52)
Low experience	812	56.55 vs 41.14	.07	1.50 (0.96-2.34)	52.91 vs 33.87	.03	1.78 (1.05-3.02)

Abbreviations: AI, artificial intelligence; BBPS, Boston bowel preparation scale; OR, odds ratio.

Further analysis was performed to examine the assistance capability of different AI systems. All AI systems showed higher assistance capability in late sessions compared with early sessions for ADR (OR, CADe: 2.75; 95% CI, 0.98-7.68 vs OR, 1.49; 95% CI, 0.87-2.56; CAQ: OR, 3.56; 95% CI, 1.89-6.72 vs OR, 1.51; 95% CI, 0.98-2.32; combination: OR, 10.40; 95% CI, 3.85-28.05 vs OR, 1.89; 95% CI, 1.12-3.21) (eTable 4 in Supplement 1). AI assistance capability in different RCTs were shown in eTable 5 in Supplement 1.

### AI Assistance Capability Over Different Hours

Among the control-c group, a significant decline in ADR was noted for hour passage of the half day with the lowest ADR in the last hour per half day at 2.99% (noon) and 0.00% (4 pm), respectively (Table 4; eFigure in Supplement 1). Correspondingly, AI systems showed the highest assistance capability in the last hour per half day (noon, 20.22% vs 2.99%; *P* = .009; OR, 8.71; 95% CI, 1.72-44.07; 4 pm, 24.32% vs 0.00%).

**Table 4.** Comparison of AI Assistance Capabilities Over Different Hours

Hour	ADR, No. (%)		*P* value	OR (95% CI)	PDR, No. (%)		*P* value	OR (95% CI)
	Unassisted	AI			Unassisted	AI		
8 am	6 (8.96)	17 (22.08)	.07	2.98 (0.91-9.76)	24 (35.82)	44 (57.14)	.02	2.63 (1.17-5.91)
9 am	17 (15.18)	63 (27.27)	.15	1.63 (0.84-3.14)	49 (43.75)	128 (55.41)	.20	1.43 (0.83-2.49)
10 am	20 (17.09)	53 (22.94)	.19	1.56 (0.81-3.01)	55 (47.01)	135 (58.44)	.12	1.54 (0.89-2.65)
11 am	11 (11.22)	53 (26.24)	.006	2.98 (1.36-6.54)	39 (39.80)	113 (55.94)	.03	1.88 (1.06-3.31)
Noon	2 (2.99)	36 (20.22)	.009	8.71 (1.72-44.07)	21 (31.34)	92 (51.69)	.03	2.23 (1.07-4.66)
1 pm	4 (8.70)	16 (15.69)	.83	1.16 (0.30-4.52)	15 (32.61)	54 (52.94)	.35	1.51 (0.64-3.53)
2 pm	2 (13.33)	8 (18.60)	.54	2.16 (0.18-26.53)	5 (33.33)	22 (51.16)	.63	1.57 (0.25-9.74)
3 pm	2 (4.55)	7 (11.86)	.49	1.96 (0.29-13.35)	12 (27.27)	25 (42.37)	.91	1.08 (0.33-3.49)
4 pm	0	9 (24.32)	NA	NA	11 (20.37)	19 (51.35)	.04	3.68 (1.07-12.67)

Abbreviations: AI, artificial intelligence; ADR, adenoma detection rate; NA, not applicable; OR, odds ratio; PDR, polyp detection rate.

### AI Assistance Capability Among Different Lesion Sizes and Locations

For adenoma detection, the AI assistance capability increase in the late sessions compared with early sessions was mainly associated with an increase in diminutive (OR, 3.52; 95% CI, 1.90-6.52 vs OR, 1.42; 95% CI, 0.96-1.08), small adenomas (OR, 7.32; 95% CI, 0.93-57.63 vs OR, 3.35; 95% CI, 1.14-9.82) and distal colon adenomas (OR, 4.51; 95% CI, 1.94-10.49 vs OR, 1.40; 95% CI, 0.89-2.23) (Table 5). With the assistance of the CADe system, more diminutive adenomas (OR, 2.42; 95% CI, 0.97-5.99 vs OR, 1.59; 95% CI, 0.95-2.68) were detected during late sessions compared with early sessions, while more small adenomas (OR, 9.20; 95% CI, 1.10-76.85 vs OR, 1.09; 95% CI, 0.26-4.63) were detected by the CAQ system. Different AI system assistance capabilities among different lesion sizes and locations are shown in eTable 6 and eTable 7 in Supplement 1.

**Table 5.** Comparison of AI Assistance Capabilities Among Different Sizes and Locations

Capability	Early session				Late session			
	No. (%)		*P* value	OR (95% CI)	No. (%)		*P* value	OR (95% CI)
	Unassisted (n = 357)	AI (n = 684)			Unassisted (n = 263)	AI (n = 476)		
Adenoma detection rate								
Diminutive (≤5 mm)	47 (13.17)	139 (20.32)	.08	1.42 (0.96-2.08)	14 (5.32)	96 (20.17)	<.001	3.52 (1.90-6.52)
Small (>5 to <10 mm)	4 (1.12)	30 (4.39)	.03	3.35 (1.14-9.82)	1 (0.38)	18 (3.78)	.06	7.32 (0.93-57.63)
Large (≥10 mm)	2 (0.56)	21 (3.07)	.03	5.16 (1.16-22.98)	1 (0.38)	12 (2.52)	.10	5.71 (0.70-46.76)
Proximal colon	22 (6.16)	85 (12.43)	.01	1.95 (1.17-3.25)	8 (3.04)	55 (11.55)	.006	3.03 (1.37-6.72)
Distal colon	29 (8.12)	93 (13.60)	.15	1.40 (0.89-2.23)	7 (2.66)	57 (11.97)	<.001	4.51 (1.94-10.49)
Polyp detection rate								
Diminutive (≤5 mm)	142 (39.78)	364 (53.22)	.01	1.47 (1.10-1.98)	80 (30.42)	238 (50.00)	.001	1.84 (1.28-2.65)
Small (>5 to <10 mm)	10 (2.80)	41 (6.00)	.06	2.00 (0.96-4.18)	6 (2.28)	27 (5.67)	.35	1.57 (0.61-4.02)
Large (≥10 mm)	3 (0.84)	28 (4.09)	.01	4.62 (1.36-15.75)	3 (1.14)	15 (3.15)	.31	1.95 (0.53-7.19)
Proximal colon	73 (20.45)	182 (26.61)	.31	1.19 (0.85-1.67)	37 (14.07)	120 (25.21)	.04	1.60 (1.02-2.51)
Distal colon	113 (31.65)	293 (42.84)	.03	1.39 (1.03-1.87)	60 (22.81)	201 (42.23)	<.001	2.00 (1.36-2.94)

Abbreviations: AI, artificial intelligence; OR, odds ratio.

## Discussion

In this cohort study, we found that later colonoscopy sessions per half day were associated with a decline in adenoma detection (early vs late, 13.73% vs 5.70%; *P* = .005; OR, 2.42; 95% CI, 1.31-4.47) during all day shift schedules. Furthermore, with the assistance of AI systems, no such association was found in both ADR (early vs late, 22.95% vs 22.06%; *P* = .78; OR, 0.96; 95% CI, 0.71-1.29) and PDR (early vs late, 55.99% vs 52.31%; *P* = .57; OR, 1.08; 95% CI, 0.83-1.40), which suggests that AI systems could overcome the time-related degradation of colonoscopy quality. To the best of our knowledge, this is the first study evaluating the association between AI systems and time of day through a prospective clinical trial. We believe that AI systems will be an effective tool for minimizing the time-related degradation of colonoscopy quality.

Extensive studies have explored the adverse outcome of later time of day on clinical performance in other domains, such as increasing omission errors and false positives among radiologists and decreasing abnormal cytology detection rates on gynecologic cytology samples among cytotechnologists.^18,19^ Radiologists reported eyestrain and blurred vision with an increased number of images read. They hold an opinion that time of day significantly impacts reading competence-related cognitive functions. This phenomenon is not only manifested for the previously mentioned clinical departments, but also for endoscopists. Korenblit et al^20^ reported a decline in sensitivity and diagnostic accuracy of endoscopic ultrasound-guided fine needle aspiration for solid pancreatic lesions with progressively later starting times of endoscopic ultrasounds, which combines the technical aspects of endoscopy, real-time interpretation of moving images, and fine needle aspiration sampling. The result was explicitly interpreted as the accumulation of fatigue due to prolonged concentration, comprehensive image review, and demands to read quickly.

Among endoscopists, increasing amounts of colonoscopies posed a sharply increased burden to endoscopists, leading to a decrease of ADR.^21^ According to a national CRC screening program, ADR reduction was associated with the second half of a session (11 am to 2 pm or 4 pm to 6 pm).^9^ Our study showed the same trend in colonoscopies without AI intervention. Due to the reliance on stereotypic movements during colonoscopies, as well as the immediate analysis of images, we proposed that increasing procedures may result in associated fatigue and distraction, which in turn leads to cognitive errors and misdiagnosis.^6,8,10,22^ Furthermore, subgroup analysis was performed to explore whether the conclusion was influenced by confounding factors, such as withdrawal time, bowel preparation, and endoscopist experience.^23,24,25^ We also found a decline in ADR in late sessions compared with early sessions among different colonoscopy settings. For these aforementioned reasons, we proposed that the accrual of endoscopist fatigue may be an independent factor of time-related degradation of colonoscopy quality.

To address the previously mentioned problem, some studies revealed that by applying a 3-hour colonoscopy shift schedule, an assigned time of 45 minutes per colonoscopy, or a schedule of 6 patients (for an esophagogastroduodenoscopy, colonoscopy, or both) per 4-hour block, the decline in ADR associated with time of day could be eliminated, suggesting that shorter shifts and fewer colonoscopy procedures may be a potential approach to overcome the time-related problem.^16,26,27^ However, the existing approach had limitations as it is not applicable to many institutions with high workloads and different working schedules. Our study proposed 2 real-time computer-aided systems based on deep learning and demonstrated that the intervention of AI systems was associated with an increase in ADR all-day, especially later sessions per half day, even in different colonoscopy settings. In the future, AI systems have the potential to ensure the quality of colonoscopies in large–workload clinical practices.

Adenomas are missed for 2 main reasons; one is failure to recognize polyps within the visual field due to cognitive limitations, and the other is the existence of blind spots due to technical defects.^28^ Furthermore, as the day progresses, repetitive stereotypic maneuvering and visual monotony could lead to decreased cognitive levels and increased medical error.^8,29^ To augment cognition, we developed a CADe system with real-time visual alerts when identifying polyps on the monitor. The ADR was significantly higher in the CADe group compared with that of the control group in our previous study. In addition, the CADe system showed higher assistance capability at late sessions compared with early sessions (OR, 2.75; 95% CI, 0.98-7.68 vs OR, 1.49; 95% CI, 0.87-2.56), which suggests that the CADe system is an ideal approach to address unrecognized polyps, especially at later sessions per half day.

However, the quality of mucosa exposure determined the limits of the CADe system, as polyps may be missed outside of the visual field. To avoid such nonvisualized lesions due to technical defects, we developed a CAQ system for colonoscopy quality improvement. Shorter mean annual withdrawal times were independently associated with lower ADR;^25^ however, endoscopists tended to slow down withdrawal speed at the end of the procedure to achieve standard time.^14^ Therefore, our CAQ system not only times the whole withdrawal process, but also monitors real-time withdrawal speed during procedures. In addition, avoiding endoscope slipping might be difficult for some endoscopists, particularly in the hepatic flexure.^30^ Accordingly, our CAQ system reminds endoscopists of blind spots caused by endoscope slipping to better expose mucosa and apply sufficient examination. Prior studies had estimated the validity of our CAQ system with significantly higher ADR in the CAQ group compared with that of the control group.^14,15^ Moreover, the CAQ system showed higher assistance capability at late sessions compared with early sessions (OR, 3.56; 95% CI, 1.89-6.72 vs OR, 1.51; 95% CI, 0.98-2.32). In addition, the combination system combining the CADe system and CAQ system showed the highest assistance capability during late sessions (OR, 10.40; 95% CI, 3.85-28.05 vs OR, 1.89; 95% CI, 1.12-3.21).

Regarding the lesion size and locations, the AI assistance capability increase during late sessions was mainly associated with an increase in diminutive, small adenomas and distal colon adenomas. With the assistance of the CADe system, more diminutive adenomas (OR, 2.42; 95% CI, 0.97-5.99 vs OR, 1.59; 95% CI, 0.95-2.68) were detected during late sessions compared with early sessions, while more small adenomas (OR, 9.20; 95% CI, 1.10-76.85 vs OR, 1.09; 95% CI, 0.26-4.63) were detected by the CAQ system. Generally, diminutive polyps are more likely to be missed within the visual field. To counter this case, the CADe system applies real-time visual alerts when identifying polyps. Additionally, the result that more small adenomas were detected with CAQ system assistance may be attributed to more adequate mucosal exposure, which resulted from low and uniform withdrawal speed and the reminder regarding blind spots.

Among the analysis of hourly detection rates in the control-c group, we found an increase in adenoma and polyp detection after the first hour of colonoscopy procedures per half day, which has been termed as the warming-up effect in previous studies,^10,16^ which is the need for endoscopists to perform a few endoscopy procedures to operate more comfortably before reaching better performance. In addition, a previous study^31^ about time of day in radiology expressed the opinion that clinician performance of the visual search task can be impacted by circadian rhythms, leading to different clinical performance during different times of the day; some also noted a decrease in performance after lunch. Since movements while performing colonoscopies are repetitive and stereotypical, this warming-up effect may result from increasing concentration due to improvement in endoscopists’ working state. On the other hand, we found the AI system showed the highest assistance capability in the last hour per half day (noon, 20.22% vs 2.99%; *P* = .009; OR, 8.71; 95% CI, 1.72-44.07; 4 pm, 24.32% vs 0.00%), which demonstrated the potential benefit of using AI to maintain high quality colonoscopy procedures.

### Limitations

There are some limitations in our study. First, the result was only validated at one center; whether the conclusion of this study is generalizable in clinical centers with different work schedules remains unknown, and further study is needed. Second, the ADR in the control-c group was lower than that in Western populations. Due to differences in environment, diet and lifestyle, the morbidity of colon polyps/adenomas could be different among regions and populations.^32^ Multiple regional samples are needed to further investigate the adaptability and effectiveness of our findings, especially regions of high morbidity of colon adenoma.

## Conclusions

In conclusion, our results suggest that later sessions per half day were associated with a decline adenoma detection. Furthermore, AI systems could eliminate the time-related degradation of colonoscopy quality. In the future, the application of AI systems has the potential to maintain high quality and homogeneity of colonoscopies and further improve endoscopist performance in large screening programs and centers with high workloads.

## Notes

Supplement 1.
eMethods.
**eFigure. **ADR/PDR at Each Hour of the Day **eTable 1. **Comparison of Detection Rates Between Early and Late Sessions **eTable 2. **Detection Rates Between Early and Late Session in Different RCTs **eTable 3. **Detection Rates Among Individual Endoscopists **eTable 4. **Comparison of AI Assistance Capabilities Between Early and Late Sessions **eTable 5. **AI Assistance Capabilities Between Early and Late Sessions in Different RCTs **eTable 6. **Different AI Systems Assistance Capabilities Among Different Sizes **eTable 7. **Different AI Systems Assistance Capabilities Among Different Locations

Supplement 2.
Data Sharing Statement
