# Supplementary material for: Assessment of the Role of Artificial Intelligence in the Association Between Time of Day and Colonoscopy Quality
Source: JAMA Netw Open. 2023 Jan 31;6(1):e2253840. doi: 10.1001/jamanetworkopen.2022.53840 (PMC9890283; doi:10.1001/jamanetworkopen.2022.53840)
Supplement: Supplement 1. — eMethods. eFigure. ADR/PDR at Each Hour of the Day eTable 1. Comparison of Detection Rates Between Early and Late Sessions eTable 2. Detection Rates Between Early and Late Session in Different RCTs eTable 3. Detection Rates Among Individual Endoscopists eTable 4. Comparison of AI Assistance Capabilities Between Early and Late Sessions eTable 5. AI Assistance Capabilities Between Early and Late Sessions in Different RCTs eTable 6. Different AI Systems Assistance Capabilities Among Different Sizes eTable 7. Different AI Systems Assistance Capabilities Among Different Locations [file jamanetwopen-e2253840-s001.pdf]

Supplemental Online Content

Lu Z, Zhang L, Yao L, et al. Assessment of the role of artificial intelligence in the association between time of day and colonoscopy quality. *JAMA Netw Open*. 2023;6(1):e2253840. doi:10.1001/jamanetworkopen.2022.53840

eMethods.
eFigure. ADR/PDR at Each Hour of the Day
eTable 1. Comparison of Detection Rates Between Early and Late Sessions
eTable 2. Detection Rates Between Early and Late Session in Different RCTs
eTable 3. Detection Rates Among Individual Endoscopists
eTable 4. Comparison of AI Assistance Capabilities Between Early and Late Sessions
eTable 5. AI Assistance Capabilities Between Early and Late Sessions in Different RCTs
eTable 6. Different AI Systems Assistance Capabilities Among Different Sizes
eTable 7. Different AI Systems Assistance Capabilities Among Different Locations

This supplemental material has been provided by the authors to give readers additional information about their work.

© 2023 Lu Z et al. *JAMA Network Open.*

eMethods

Intervention

In the RCT 1, patients were assigned to either control group (henceforth called control-1 group) or CAQ group (henceforth called CAQ-1 group). In the CAQ-1 group, a real-time quality improvement system was constructed to assist endoscopists, which could achieve real-time withdrawal speed, colonoscopy intubation, and withdrawal time monitoring, and remind endoscopists of blind spots caused by endoscope slipping. To obtain the withdrawal speed, the similarity among continuous colonoscopy frames was calculated using the perceptual hash algorithm. The value of less than 40 was set as safe withdrawal speed, 40-44 as t alarm withdrawal speed and more than 44 as dangerous withdrawal speed after validation. There's no intervention in the control-a group. The results showed that ADR was significantly higher in the CAQ group than in the control group (16% vs 8%, P=0.001).

In the RCT 2, four groups were evaluated: control group (henceforth called control-2 group), CAQ group (henceforth called CAQ-2 group), CADe group, combination group. The interventions in CAQ-2 group were the same as those in CAQ-1 group. In the CADe group, a real-time polyp detection system was constructed to assist endoscopists. The combination group included both interventions from CAQ group and CADe group. There's no intervention in the control-2 group. The results showed that the ADR was significantly higher in the CADe group (21.27% vs 14.76%, P=0.045), CAQ-2 group (24.54% vs 14.76%, P=0.004) and combination group (30.60% vs 14.76%, P<0.001) compared with the control-2 group, respectively.

Definition

ADR was defined as the proportion of patients having at least one histologically confirmed adenoma during colonoscopy. Polyp detection rate (PDR) was defined as the proportion of patients who have at least one polyp detected during colonoscopy. We defined proximal colon as cecum, ascending colon, hepatic flexure or transverse colon, distal colon as splenic flexure, descending colon, sigmoid colon or rectum. Advanced adenoma was defined as adenoma with one of the following features: (1) ≥ 1 cm as documented by the endoscopist, (2) with villous architecture on histology or (3) with high-grade dysplasia.

© 2023 Lu Z et al. *JAMA Network Open.*

eFigure. ADR/PDR at Each Hour of the Day

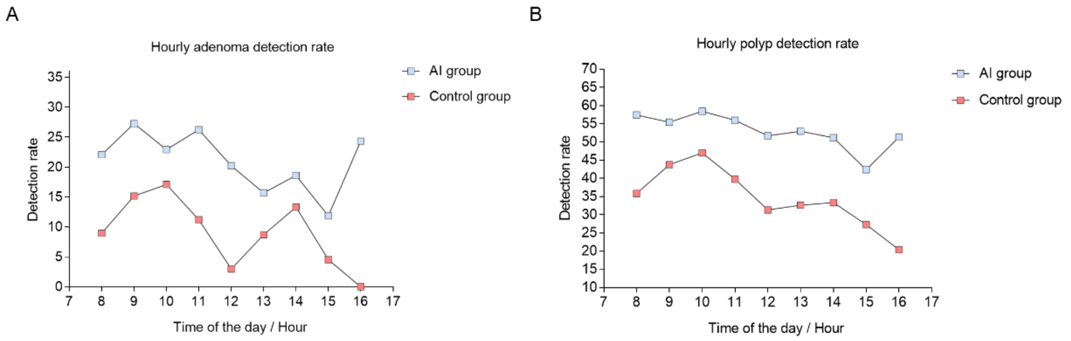

A: Adenoma detection rate (ADR) and B: polyp detection rate (PDR) in each hour

© 2023 Lu Z et al. *JAMA Network Open.*

eTable 1. Comparison of Detection Rates Between Early and Late Sessions				
	Early session	Late session	P	OR (95% CI)
**Advanced adenoma detection rate**				
Control-c group	5(1.40%)	3(1.14%)	.52	1.66(0.36-7.71)
AI group	26(3.80%)	17(3.57%)	.73	1.12(0.59-2.13)
CADe group	4(2.41%)	1(0.98%)	.38	2.97(0.27-33.08)
CAQ-c group	11(3.15%)	13(4.73%)	.34	0.66(0.28-1.55)
Combination group	11(6.51%)	3(3.03%)	.27	2.15(0.56-8.27)
**Adenomas detected per colonoscopy, mean(SD)**				
Control-c group	0.15(0.37)	0.06(0.25	.01	2.10(1.16-3.80)
AI group	0.27(0.53)	0.25(0.49)	.99	1.00(0.79-1.27)
CADe group	0.28(0.52)	0.18(0.43)	.10	1.63(0.91-2.91)
CAQ-c group	0.21(0.44)	0.22(0.47)	.69	0.93(0.66-1.32)
Combination group	0.36(0.69)	0.39(0.59)	.62	0.90(0.60-1.35)
**Polyps detected per colonoscopy, mean(SD)**				
Control-c group	1.09(1.92)	0.77(1.57)	.40	1.08(0.91-1.29)
AI group	1.61(2.47)	1.45(2.21)	.42	1.04(0.95-1.15)
CADe group	1.63(2.35)	1.49(2.30)	.92	1.01(0.82-1.25)
CAQ-c group	1.28(1.91)	1.33(2.13)	.46	0.95(0.83-1.09)
Combination group	2.26(3.35)	1.72(2.32)	.002	1.34(1.12-1.61)

eTable 2. Detection Rates Between Early and Late Session in Different RCTs								
	ADR		P	OR (95% CI)	PDR		P	OR (95% CI)
	Early session	Late session			Early session	Late session		
RCT 1								
Control-1 group	17(9.83%)	10(5.68%)	.25	1.64(0.70-3.81)	62(35.84%)	56(31.82%)	.67	1.11(0.68-1.82)
CAQ-1group	24(13.71%)	34(18.89%)	.16	0.65(0.36-1.18)	74(42.29%)	92(51.11%)	.09	0.68(0.43-1.06)
RCT 2								
Control-2 group	32(17.39%)	5(5.75%)	.02	3.22(1.20-8.70)	86(46.74%)	27(31.03%)	.04	1.83(1.02-3.28)
CAQ-2 group	44(25.29%)	21(22.11%)	.41	1.32(0.69-2.52)	97(55.75%)	48(50.53%)	.39	1.29(0.73-2.28)
CADe group	42(25.30%)	15(14.71%)	.08	1.97(0.93-4.17)	102(61.45%)	47(46.08%)	.12	1.58(0.89-2.81)
Combination group	47(27.81%)	35(35.35%)	.13	0.63(0.34-1.15)	110(65.09%)	62(62.63%)	.65	1.15(0.63-2.08)

© 2023 Lu Z et al. *JAMA Network Open.*

eTable 3. Detection Rates Among Individual Endoscopists												
	Endoscopist 1						Endoscopist 2					
	Control-c (n=144)	P	AI (n=254)	P	Total (n=398)	P	Control-c (n=114)	P	AI (n=344)	P	Total (n=458)	P
**Adenoma detection rate**												
AI group	-	-	-	-	49(19.29%)	<.05	-	-	-	-	100(29.07%)	<.001
Control-c group	-	-	-	-	13(9.03%)		-	-	-	-	13(11.40%)	
**Time slot 1**												
Early session	9(12.16%)	.20	22(17.32%)	.24	31(15.42%)	.72	12(16.00%)	.05	73(31.33%)	.16	85(27.60%)	.05
Late session	4(5.71%)		27(21.26%)		31(15.74%)		1(2.56%)		27(24.32%)		28(18.67%)	
**Time slot 2**												
AM	11(11.34%)	.44	38(20.21%)	.60	49(17.19%)	.27	11(10.68%)	.67	87(30.53%)	.08	98(25.26%)	.12
PM	2(4.26%)		11(16.67%)		13(11.50%)		2(18.18%)		13(22.03%)		15(21.43%)	
**Withdrawal time**												
≥ 6min	7(11.11%)	.66	43(20.19%)	.57	50(18.12%)	.42	9(9.68%)	.07	98(30.43%)	<.05	107(25.78%)	.48
< 6min	6(7.41%)		6(14.63%)		12(9.84%)		4(19.05%)		2(9.09%)		6(13.95%)	
**BBPS**												
≥2 in all segments	12(9.16%)	.51	45(19.74%)	.44	57(15.88%)	.40	10(11.36%)	.92	81(28.93%)	.55	91(24.73%)	.85
<2 in any segment	1(7.69%)		4(15.38%)		5(12.82%)		3(11.54%)		19(29.69%)		22(24.44%)	
**Polyp detection rate**												
AI group	-	-	-	-	142(55.91%)	.007	-	-	-	-	198(57.56%)	<.001
Control group	-	-	-	-	49(34.03%)		-	-	-	-	41(35.96%)	
**Time slot 1**												
Early session	24(32.43%)	.37	73(57.48%)	.90	97(48.26%)	.96	31(41.33%)	.10	137(58.80%)	.87	168(54.55%)	.37
Late session	25(35.71%)		69(54.33%)		94(47.72%)		10(25.64%)		61(54.95%)		71(47.33%)	
**Time slot 2**												
AM	38(39.18%)	.02	103(54.79%)	.19	141(49.47%)	.74	37(35.92%)	.94	167(58.60%)	.31	204(52.58%)	.25
PM	11(23.40%)		39(59.09%)		50(44.25%)		4(36.36%)		31(52.54%)		35(50.00%)	

© 2023 Lu Z et al. *JAMA Network Open.*

**Withdrawal time**												
≥ 6min	29(46.03%)	.005	124(58.22%)	<.05	153(55.43%)	.01	34(36.56%)	.76	193(59.94%)	.003	227(54.70%)	.04
< 6min	20(24.69%)		18(43.90%)		38(31.15%)		7(33.33%)		5(22.73%)		12(27.91%)	
**BBPS**												
≥2 in all segments	42(32.06%)	.18	131(57.46%)	.18	173(48.19%)	.50	28(31.82%)	.24	166(59.29%)	.36	194(52.72%)	.76
<2 in any segment	7(53.85%)		11(42.31%)		18(46.15%)		13(50.00%)		32(50.00%)		45(50.00%)	

(continued)

© 2023 Lu Z et al. *JAMA Network Open.*

	Endoscopist 3						Endoscopist 4					
	Control-c (n=102)	P	AI (n=266)	P	Total (n=368)	P	Control-c (n=97)	P	AI (n=87)	P	Total (n=184)	P
**Adenoma detection rate**												
AI group	-	-	-	-	64(24.06%)	.07	-	-	-	-	18(20.69%)	.42
Control-c group	-	-	-	-	14(13.73%)		-	-	-	-	14(14.43%)	
**Time slot 1**												
Early session	13(17.33%)	.17	38(22.62%)	.47	51(20.99%)	.89	9(16.07%)	.67	6(12.50%)	.04	15(14.42%)	.12
Late session	1(3.70%)		26(26.53%)		27(21.60%)		5(12.20%)		12(30.77%)		17(21.25%)	
**Time slot 2**												
AM	13(14.29%)	.92	57(23.65%)	.64	70(21.08%)	.81	13(17.11%)	.13	15(21.74%)	.32	28(19.31%)	.08
PM	1(9.09%)		7(28.00%)		8(22.22%)		1(4.76%)		3(16.67%)		4(10.26%)	
**Withdrawal time**												
≥ 6min	14(17.72%)	NA	60(26.67%)	.03	74(24.34%)	.004	5(22.73%)	.17	7(21.21%)	.93	12(21.82%)	.47
< 6min	0(0.00%)		4(9.76%)		4(6.25%)		9(12.00%)		11(20.37%)		20(15.50%)	
**BBPS**												
≥2 in all segments	13(13.83%)	.47	55(23.61%)	.82	68(20.80%)	.91	14(15.22%)	NA	17(20.99%)	.70	31(17.92%)	.48
<2 in any segment	1(12.50%)		9(27.27%)		10(24.39%)		0(0.00%)		1(16.67%)		1(9.09%)	
**Polyp detection rate**												
AI group	-	-	-	-	153(57.52%)	.04	-	-	-	-	41(47.13%)	.66
Control group	-	-	-	-	45(44.12%)		-	-	-	-	43(44.33%)	
**Time slot 1**												
Early session	38(50.67%)	.09	99(58.93%)	.42	137(56.38%)	.17	29(51.79%)	.49	19(39.58%)	.17	48(46.15%)	.64
Late session	7(25.93%)		54(55.10%)		61(48.80%)		14(34.15%)		22(56.41%)		36(45.00%)	
**Time slot 2**												
AM	39(42.86%)	.32	141(58.51%)	.30	180(54.22%)	.71	38(50.00%)	.06	33(47.83%)	.55	71(48.97%)	.10
PM	6(54.55%)		12(48.00%)		18(50.00%)		5(23.81%)		8(44.44%)		13(33.33%)	
**Withdrawal time**												
≥ 6min	36(45.57%)	.57	139(61.78%)	.002	175(57.57%)	.009	13(59.09%)	.82	14(42.42%)	.18	27(49.09%)	.55
< 6min	9(39.13%)		14(34.15%)		23(35.94%)		30(40.00%)		27(50.00%)		57(44.19%)	
**BBPS**												
≥2 in all segments	42(44.68%)	.80	135(57.94%)	.34	177(54.13%)	.47	40(43.48%)	.79	39(48.15%)	.17	79(45.66%)	.21
<2 in any segment	3(37.50%)		18(54.55%)		21(51.22%)		3(60.00%)		2(33.33%)		5(45.45%)	

© 2023 Lu Z et al. *JAMA Network Open.*

eTable 4. Comparison of AI Assistance Capabilities Between Early and Late Sessions						
	Early session			Late session		
	% vs %	P	OR (95% CI)	% vs %	P	OR (95% CI)
**Adenoma detection rate**						
AI VS Control-c	22.95% vs 13.73%	.01	1.60(1.10-2.34)	22.06% vs 5.70%	<.001	3.81(2.10-6.91)
CADe VS Control-c	25.30% vs 13.73%	.15	1.49(0.87-2.56)	14.71% vs 5.70%	.05	2.75(0.98-7.68)
CAQ-c VS Control-c	19.48% vs 13.73%	.06	1.51(0.98-2.32)	20.00% vs 5.70%	<.001	3.56(1.89-6.72)
Combination VS Control-c	27.81% vs 13.73%	.02	1.89(1.12-3.21)	35.35% vs 5.70%	<.001	10.40(3.85-28.05)
**Polyp detection rate**						
AI VS Control-c	55.99% vs 41.46%	.005	1.53(1.14-2.06)	52.31% vs 31.56%	.001	1.90(1.32-2.73)
CADe VS Control-c	61.45% vs 41.46%	.02	1.77(1.12-2.81)	46.08% vs 31.56%	.18	1.52(0.83-2.79)
CAQ-c VS Control-c	49.00% vs 41.46%	.10	1.33(0.95-1.86)	50.91% vs 31.56%	.002	1.91(1.28-2.85)
Combination VS CONTROL-c	65.09% vs 41.46%	<.001	2.50(1.56-4.00)	62.63% vs 31.56%	.002	2.75(1.46-5.18)
**Advanced adenoma detection rate**						
AI VS Control-c	3.80% vs 1.40%	.70	1.33(0.30-5.85)	3.57% vs 1.14%	.10	3.01(0.81-11.15)
CADe VS Control-c	2.41% 1.40%	.71	1.30(0.32-5.35)	0.98% vs 1.14%	.99	NA
CAQ-c VS Control-c	3.15% vs 1.40%	.23	1.97(0.65-5.96)	4.73% vs 1.14%	.07	3.48(0.92-13.14)
Combination VS Control-c	6.51% vs 1.40%	.10	2.60(0.83-8.18)	3.03% vs 1.14%	.99	NA
**Adenomas detected per colonoscopy, mean(SD)**						
AI VS Control-c	0.27(0.53) vs 0.15(0.37)	.009	1.52(1.11-2.08)	0.25(0.49) vs 0.06(0.25)	<.001	3.12(1.80-5.36)
CADe VS Control-c	0.28(0.52) vs 0.15(0.37)	.09	1.45(0.94-2.23)	0.18(0.43) vs 0.06(0.25)	.04	2.51(1.04-6.06)
CAQ-c VS Control-c	0.21(0.44) vs 0.15(0.37)	.06	1.42(0.99-2.04)	0.22(0.47) vs 0.06(0.25)	<.001	3.07(1.73-5.46)
Combination VS CONTROL-c	0.36(0.69) vs 0.15(0.37)	.004	1.83(1.21-2.76)	0.39(0.59) vs 0.06(0.25)	<.001	4.95(2.27-10.76)
**Polyps detected per colonoscopy, mean(SD)**						
AI VS Control-c	1.61(2.47) vs 1.09(1.92)	<.001	127(1.12-1.43)	1.45(2.21) vs 0.77(1.57)	.001	1.32(1.12-1.56)
CADe VS Control-c	1.63(2.35) vs 1.09(1.92)	.42	1.07(0.91-1.27)	1.49(2.30) vs 0.77(1.57)	.05	1.28(1.00-1.64)
CAQ-c VS Control-c	1.28(1.91) vs 1.09(1.92)	.08	1.13(0.99-1.30)	1.33(2.13) vs 0.77(1.57)	.002	1.32(1.10-1.58)

© 2023 Lu Z et al. *JAMA Network Open.*

Combination VS Control-c	2.26(3.35) vs 1.09(1.92)	<.001	1.60(1.37-1.87)	1.72(2.32) vs 0.77(1.57)	.20	1.17(0.92-1.50)

© 2023 Lu Z et al. *JAMA Network Open.*

eTable 5. AI Assistance Capabilities Between Early and Late Sessions in Different RCTs

	Early session		Late session	
	P	OR (95% CI)	P	OR (95% CI)
**Adenoma detection rate**				
Control-1 VS CAQ-1	.37	1.41(0.67-2.94)	.003	3.30(1.49-7.32)
Control-2 VS CAQ-2	<.05	1.73(1.01-2.98)	.005	4.74(1.60-14.11)
Control-2 VS CADe	.15	1.51(0.87-2.64)	.05	2.97(0.98-8.99)
Control-2 VS Combination	.03	1.82(1.07-3.10)	<.001	9.92(3.24-30.41)
**Polyp detection rate**				
Control-1 VS CAQ-1	.77	1.08(0.65-1.79)	.04	1.68(1.01-2.77)
Control-2 VS CAQ-2	.04	1.63(1.02-2.59)	.02	2.33(1.18-4.62)
Control-2 VS CADe	.02	1.77(1.11-2.82)	.03	2.05(1.05-3.98)
Control-2 VS Combination	<.001	2.39(1.48-3.85)	<.001	4.04(1.97-8.32)

© 2023 Lu Z et al. *JAMA Network Open.*

eTable 6. Different AI Systems Assistance Capabilities Among Different Sizes								
	Early session				Late session			
	Control-c group	AI system	P	OR (95% CI)	Control-c group	AI system	P	OR (95% CI)
**ADR**								
CADe system								
Diminutive (≤5 mm)	47(13.17%)	39(23.49%)	.08	1.59(0.95-2.68)	14(5.32%)	16(15.69%)	.06	2.42(0.97-5.99)
Small (>5 to <10 mm)	4(1.12%)	10(6.02%)	.009	5.75(1.56-21.25)	1(0.38%)	3(2.94%)	.30	4.40(0.27-70.83)
Large (≥10mm)	2(0.56%)	2(1.20%)	.64	1.62(0.21-12.37)	1(0.38%)	0(0.00%)	NA	NA
CAQ-c system								
Diminutive (≤5 mm)	47(13.17%)	62(17.77%)	.22	1.31(0.85-2.02)	14(5.32%)	46(16.73%)	.001	3.08(1.60-5.94)
Small (>5 to <10 mm)	4(1.12%)	5(1.43%)	.91	1.09(0.26-4.63)	1(0.38%)	10(3.64%)	.04	9.20(1.10-76.85)
Large (≥10mm)	2(0.56%)	9(2.58%)	<.05	4.89(1.01-23.77)	1(0.38%)	9(3.27%)	<.05	8.76(1.05-72.81)
Combination system								
Diminutive (≤5 mm)	47(13.17%)	38(22.49%)	.10	1.57(0.93-2.67)	14(5.32%)	34(34.34%)	<.001	7.04(3.20-15.45)
Small (>5 to <10 mm)	4(1.12%)	15(8.88%)	.003	6.66(1.89-23.48)	1(0.38%)	5(5.05%)	.05	NA
Large (≥10mm)	2(0.56%)	10(5.92%)	.02	7.29(1.47-36.04)	1(0.38%)	3(3.03%)	.14	NA
**PDR**								
CADe system								
Diminutive (≤5 mm)	142(39.78%)	97(58.43%)	.02	1.66(1.08-2.57)	80(30.42%)	45(44.12%)	.26	1.40(0.78-2.49)
Small (>5 to <10 mm)	10(2.80%)	11(6.63%)	.08	2.35(0.90-6.13)	6(2.28%)	6(5.88%)	.94	1.05(0.30-3.68)
Large (≥10mm)	3(0.84%)	5(3.01%)	.15	3.20(0.67-15.27)	3(1.14)	1(0.98%)	.91	0.86(0.06-11.84)
CAQ-c system								
Diminutive (≤5 mm)	142(39.78%)	164(46.99%)	.18	1.26(0.90-1.75)	80(30.42%)	133(48.36%)	.003	1.81(1.22-2.68)
Small (>5 to <10 mm)	10(2.80%)	9(2.58%)	.88	0.93(0.35-2.44)	6(2.28%)	16(5.83%)	.27	1.78(0.64-4.91)
Large (≥10mm)	3(0.84%)	11(3.15%)	.04	4.11(1.10-15.38)	3(1.14)	10(3.64%)	.11	3.01(0.77-11.76)
Combination system								
Diminutive (≤5 mm)	142(39.78%)	103(60.95%)	.001	2.17(1.39-3.38)	80(30.42%)	60(60.61%)	.001	2.71(1.50-4.89)
Small (>5 to <10 mm)	10(2.80%)	21(12.43%)	.001	4.39(1.81-10.66)	6(2.28%)	5(5.05%)	.79	0.82(0.20-3.34)

© 2023 Lu Z et al. *JAMA Network Open.*

Large (≥10mm)	3(0.84%)	12(7.10%)	.003	8.22(2.00-33.76)	3(1.14)	4(4.04%)	.41	2.09(0.37-11.87)

eTable 7. Different AI Systems Assistance Capabilities Among Different Locations

	Early session				Late session			
	Control-c group	AI system	P	OR (95% CI)	Control-c group	AI system	P	OR (95% CI)
**ADR**								
CADe system								
Proximal Colon	22(6.16%)	21(12.65%)	.12	1.72(0.87-3.41)	8(3.04%)	10(9.80%)	.07	3.06(0.93-10.09)
Distal Colon	29(8.12%)	26(15.66%)	.09	1.70(0.91-3.15)	7(2.66%)	6(5.88%)	.41	2.20(1.80-7.24)
CAQ-c system								
Proximal Colon	22(6.16%)	41(11.75%)	.01	2.02(1.15-3.53)	8(3.04%)	26(9.45%)	.02	2.81(1.21-6.54)
Distal Colon	29(8.12%)	35(10.03%)	.71	1.11(0.65-1.89)	7(2.66%)	34(12.36%)	<.001	4.77(1.99-11.46)
Combination system								
Proximal Colon	22(6.16%)	23(13.61%)	.04	2.10(1.05-4.20)	8(3.04%)	19(19.19%)	<.001	6.63(2.36-18.62)
Distal Colon	29(8.12%)	32(18.93%)	.02	2.10(1.16-3.81)	7(2.66%)	17(17.17%)	.002	5.73(1.93-17.07)
**PDR**								
CADe system								
Proximal Colon	73(20.45%)	46(27.71%)	.52	1.17(0.72-1.89)	37(14.07%)	25(24.51%)	.13	1.73(0.85-3.50)
Distal Colon	113(31.65%)	82(49.40%)	.03	1.62(1.05-2.50)	60(22.81%)	37(36.27%)	.16	1.55(0.85-2.82)
CAQ-c system								
Proximal Colon	73(20.45%)	86(24.64%)	.34	1.21(0.82-1.77)	37(14.07%)	64(23.27%)	.09	1.52(0.93-2.47)
Distal Colon	113(31.65%)	122(34.96%)	.65	1.08(0.77-1.52)	60(22.81%)	114(41.45%)	.001	2.00(1.32-3.03)
Combination system								
Proximal Colon	73(20.45%)	50(29.59%)	.13	1.45(0.89-2.35)	37(14.07%)	31(31.31%)	.006	2.59(1.32-5.09)
Distal Colon	113(31.65%)	8952.66%)	.001	2.07(1.34-3.20)	60(22.81%)	50(50.51%)	.003	2.45(1.35-4.46)

© 2023 Lu Z et al. *JAMA Network Open.*
