# Supplementary material for: Assessment of the Role of Artificial Intelligence in the Association Between Time of Day and Colonoscopy Quality
Source: JAMA Netw Open. 2023 Jan 31;6(1):e2253840. doi: 10.1001/jamanetworkopen.2022.53840 (PMC9890283; doi:10.1001/jamanetworkopen.2022.53840)
Supplement: Supplement 2. — Data Sharing Statement [file jamanetwopen-e2253840-s002.pdf]

Data Sharing Statement

Lu. Assessment of the Role of Artificial Intelligence in the Association Between Time of Day and Colonoscopy Quality. *JAMA Netw Open*. Published January 31, 2023. doi:10.1001/jamanetworkopen.2022.53840

Data
Data available: No
